# Effective detection of rare variants in pooled DNA samples using cross-pool tail-curve analysis

**DOI:** 10.1186/gb-2011-12-s1-p45

**Published:** 2011-09-19

**Authors:** Tejasvi S Niranjan, Abby Adamczyk, Hector Corrada Bravo, Margaret A Taub, Sarah J Wheelan, Rafael Irizarry, Tao Wang

**Affiliations:** 1 McKusick-Nathans Institute of Genetic Medicine and Department of Pediatrics, Johns Hopkins University School of Medicine, Baltimore, MD 21205, USA; 2 Predoctoral Training Program in Human Genetics, Johns Hopkins University School of Medicine, Baltimore, MD 21205, USA; 3 Center for Bioinformatics and Computational Biology, Department of Computer Science, University of Maryland, College Park, MD 20742, USA; 4 Present address: Center for Bioinformatics and Computational Biology, Department of Computer Science, University of Maryland, College Park, MD 20742, USA; 5 Department of Biostatistics, Bloomberg School of Public Health, Johns Hopkins University, Baltimore, MD 21205, USA; 6 Department of Oncology, Johns Hopkins University School of Medicine, Baltimore, MD 21287, USA

## 

Rare genetic variants of large effect may confer a substantial genetic risk for common diseases and complex traits. There is considerable interest in sequencing limited genomic regions such as candidate genes and target regions identified by genetic linkage and/or association studies. Next-generation sequencing of pooled DNA samples is an efficient way to identify rare variants in large sample sets. Although sample pooling can reduce the labor and cost of sequencing, it also reduces the sensitivity and specificity for effective and reliable identification of rare variants. It remains a challenge to solve these problems using the available computational genomics tools. We have developed an effective Illumina-based sequencing strategy using pooled samples and have optimized a novel base-calling algorithm, Srfim, and a variant-calling algorithm, SERVIC^4^E (Sensitive Rare Variant Identification by Cross-pool Cluster, Continuity & Tail-Curve Evaluation). SERVIC^4^E analyzes base composition by cycle or tail-curves across sample pools and employs multiple filtering strategies, including quality and continuity cluster analysis, average quality filtering, tail-curve filtering and error proximity filtering, to accurately identify rare sequence variants. We validated these algorithms using two independent Illumina sequence datasets generated from different pool sizes, read lengths and sequencing chemistries. Using these programs, we identified 32 coding variants, including 14 present only once over 24 exon-containing regions in one sample cohort (*n* = 480), and 41 coding variants, including 16 present only once in the same regions in an unrelated cohort (*n* = 480). Validation of these variants by Sanger sequencing revealed an excellent combination of sensitivity (97.8% and 96.4%) and specificity (84.9% and 93.8%) for variant detection in pooled samples from both cohorts, respectively. Data from these studies showed that our algorithms compare favorably with the available programs, including SAMtools, SNPSeeker, CRISP and Syzygy, for the effective and reliable detection of rare variants in pooled samples.

